# Supplementary material for: Towards an Image-Informed Mathematical Model of In Vivo Response to Fractionated Radiation Therapy
Source: Cancers (Basel). 2021 Apr 7;13(8):1765. doi: 10.3390/cancers13081765 (PMC8067722; doi:10.3390/cancers13081765)
Supplement: Supplementary file 1 [file cancers-13-01765-s001.pdf]

Supplemental material: Towards an image-informed mathematical model of in vivo response to fractionated radiation therapy

David A. Hormuth, II[1-2,*], Angela M. Jarrett[1-2], Tessa Davis[3], and Thomas E. Yankeelov[1-6]

[1] Oden Institute for Computational Engineering and Sciences, The University of Texas at Austin; Austin, TX 78712; David.hormuth@utexas.edu (D.A.H.), ajarrett@utexas.edu (A.M.J.),Thomas.yankeelov@utexas.edu (T.E.Y.)
[2] Livestrong Cancer Institutes, The University of Texas at Austin; Austin, TX 78712;
[3] Department of Biomedical Engineering, The University of Texas at Austin; Austin, TX, 78712; tessa@austin.utexas.edu (T.D.)
[4] Department of Diagnostic Medicine, The University of Texas at Austin; Austin, TX, 78712
[5] Department of Oncology, The University of Texas at Austin; Austin, TX, 78712
[6] Department of Imaging Physics, The University of Texas at MD Anderson Cancer Center; Houston, TX, 77030
* Correspondence: david.hormuth@utexas.edu;

S.1. Tumor segmentation

Tumor regions of interest were segmented using a semi-automatic approach by an imaging scientist with over ten years of experience in segmenting contrast-enhancing lesions in murine models of glioma. The segmented tumor consisted of the enhancing lesion on a post-contrast T_1-weighted MRI (from the DCE-MRI dataset). First, a region of interest is manually drawn around the contrast-enhancing lesion. Second, a k-means clustering in MATLAB R2020a is used to identify voxels that are enhancing and non-enhancing within the region of interest. Third, *imfill* in MATLAB R2020a is used to fill in holes within the regions identified as enhancing tissue. Finally, the k-means segmented tumor is visually inspected before proceeding with modeling. The robustness of this approach was evaluated in an *in silico* study where noise was added from a normal distribution (equivalent to an SNR of 20 and the SNR of the image used for segmentation) to each animal's day 0 post-contrast T_1-weighted MRI to generate 100 unique imaging volumes which were then segmented using the semi-automatic approach above. We then calculated the variability in volume estimates and the degree of spatial overlap using the standard error and Dice correlation coefficient, respectively. Results are shown below in Supplemental Table 1. We observed that this semi-automatic approach is robust to the noise level (SNR of 20) observed in the image used for segmentation resulting in a standard error of less than 0.26 mm^3 and Dice correlation coefficients greater than 0.91 for all animals.

Table S1. Standard error in segmented volume

Animal	Standard error (mm^3)	Dice correlation coefficient
1	0.26	0.91
2	0.24	0.92
3	0.13	0.97
4	0.16	0.96
5	0.21	0.92
6	0.10	0.97
7	0.14	0.96

S.2. Parameter calibration bounds

Model parameters during calibration are bounded as shown in Supplemental Table 2. The diffusion coefficients are bounded to maintain numerical stability, where other parameters are bounded by their definitions (i.e., volume fractions) or by 10. For parameters with an upper bound of 10, this value was sufficiently high to ensure model parameters did not get stuck at an upper bound.

Table S2. Parameter ranges used for model calibration

Parameter or variable	Parameter range	
$k_{p,T,0}$ (day^{-1})	0	10
$kd_{,T,0}$ (day^{-1})	0	10
$D_{T,0}$ ($\mu m^2\ day^{-1}$)	0	7.5×10^5
$D_{V,0}$ ($\mu m^2\ day^{-1}$)	0	7.5×10^5
$\phi_{V,thresh}$	0	1
θ_{max}	0	1
$k_{p,V}$ (day^{-1})	0	10
$k_{d,V}$ (day^{-1})	0	10
SF	0	1
α_1	0	10

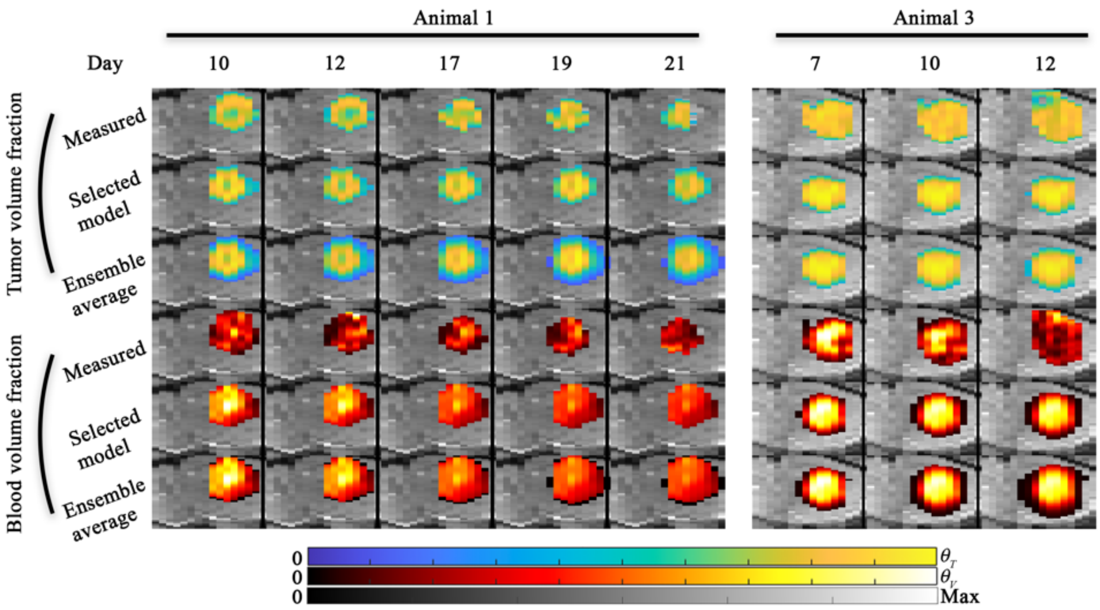

Figure S1. Model predictions (scenario 2) for animal 1 and 3 (Sagittal plane). Model predictions of tumor and blood volume fractions at the central slice are shown for animal 1 and animal 3. For both animals, the model predictions from the selected model and the ensemble average are shown. The bottom two rows show the T_2-weighted and post-contrast T_1-weighted MRI. For animal 1, both the selected and ensemble average model predict the areas of intratumor heterogeneity in the tumor volume predictions. Blood volume predictions tended to predict increased blood volume in the interior of the tumor relative to the periphery as observed. For animal 3, the observed tumor is more homogeneous (compared to animal 1) and the model is able to predict this distribution. Additional, both models predict a higher blood volume fraction towards the interior of the tumor at the final time point which is not present in the measured blood volume fraction. For animal 1, tumor growth was predicted from day 10 to 21 and resulted in -1.0% and 261% error in tumor volume predictions for the selected and ensemble models, respectively. For animal 3, both the selected and ensemble average model resulted in < 4.6% error in tumor volume predictions.

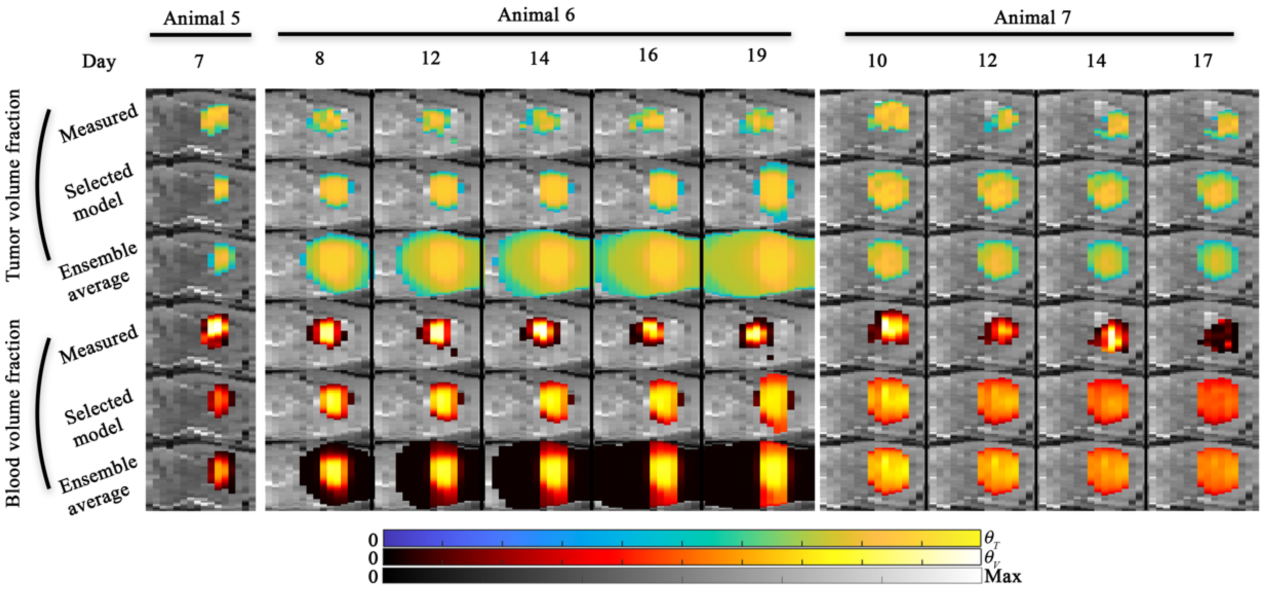

Figure S2. Model predictions (scenario 2) for animal 5, 6, and 7 (Sagittal plane). Model predictions of tumor and blood volume fractions at the central slice are shown for animal 5, 6, and 7. For all animals, the

model predictions from the selected model and the ensemble average are shown. The bottom two rows show the T_2-weighted and post-contrast T_1-weighted MRI. For animal 5, only one prediction time point was available and both the selected and ensemble average model resulted in < 8.7% error in tumor volume predictions. For animal 6, tumor growth was predicted from day 8 to 19 and resulted in 16.0% and 521% error in tumor volume predictions for the selected and ensemble models, respectively. For animal 7, four prediction time points were available and both the selected and ensemble average model resulted in < 21.5% in tumor volume predictions across all time points.

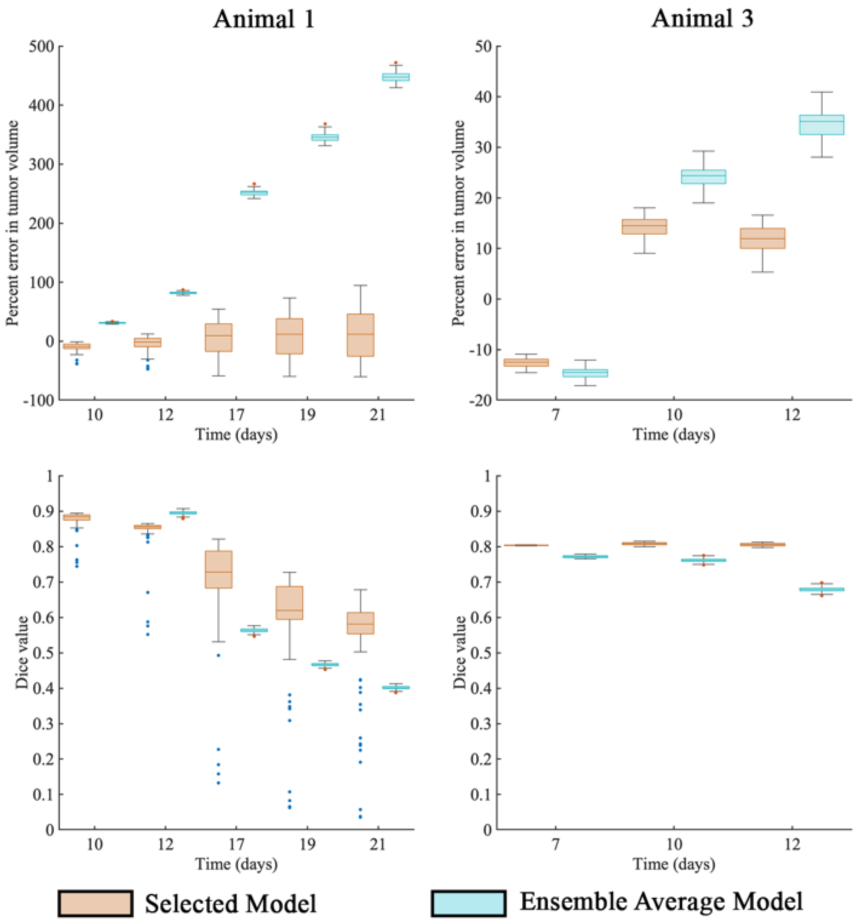

Figure S3. Error for tumor growth predictions for animals 1 and 3. The level of error in tumor volume predictions and spatial agreement of tumor volumes is assessed using the percent error in tumor volume (top row) and Dice correlation coefficient (bottom row). Results for animals 1 (left column) and 3 (right column) are shown for the selected model (orange bars) and ensemble average model (blue bars) predictions. For animal 1, the Dice value generally decreased for both the selected and ensemble average model. For animal 3, the Dice value did not decrease as much animal 1 for the selected model, while the ensemble average model did decrease over time.

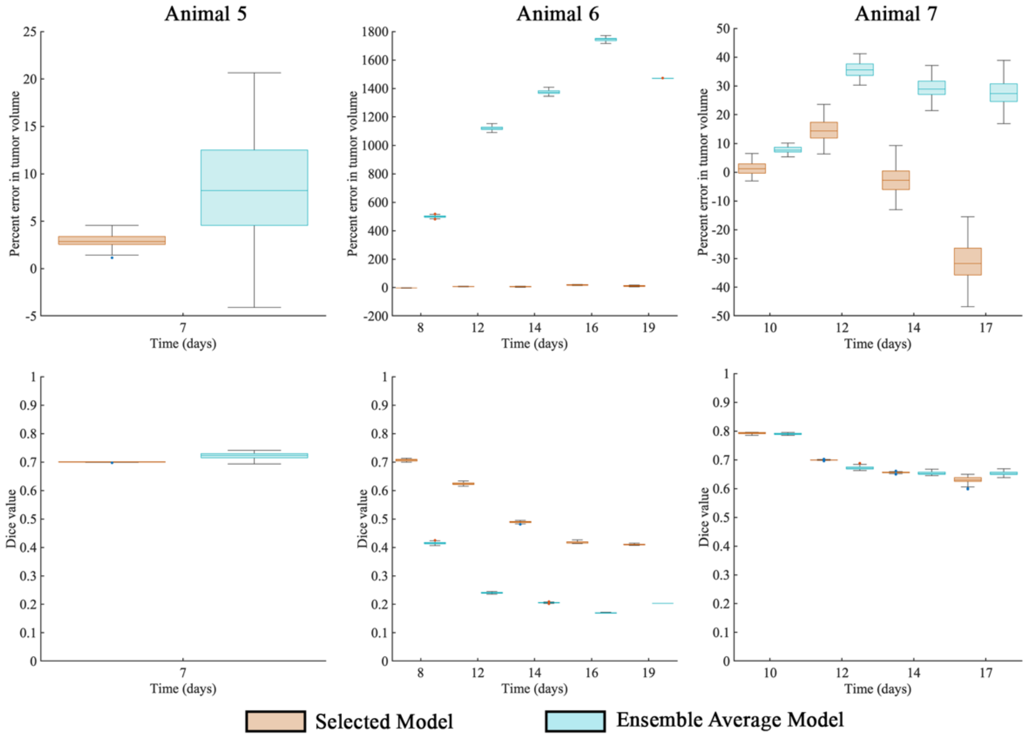

Figure S4. Error for tumor growth predictions for animals 5, 6, and 7. The level of error in tumor volume predictions and spatial agreement of tumor volumes is assessed using the percent error in tumor volume (top row) and Dice correlation coefficient (bottom row). Results for animals 5 (left column), 6 (middle column), and 7 (right column) are shown for the selected model (orange bars) and ensemble average model (blue bars) predictions. For both animals 6 and 7, the Dice value generally decreased the further out predictions were made. For animal 6, statistically significant lower Dice values were observed for the ensemble average model compared to the selected model.
